# Destructive and topical treatments of skin lesions in organ transplant recipients and relation to skin cancer

**DOI:** 10.1007/s00403-020-02136-4

**Published:** 2020-09-05

**Authors:** Adele C. Green, Mandy Way, Mariella Oster, Elsemieke I. Plasmeijer, Zainab Jiyad, Peter O’Rourke, Kyoko Miura, Scott Campbell, Nicole Isbel, Daniel C. Chambers, Peter Hopkins, Lisa E. Ferguson, Marcia Batista Davis, David C. Whiteman, H. Peter Soyer, Louise Marquart

**Affiliations:** 1 Population Health Department, QIMR Berghofer Medical Research Institute, Brisbane, QLD 4006 Australia; 2 CRUK Manchester Institute and Faculty of Biology, Medicine and Health, Manchester Academic Health Sciences Centre, University of Manchester, Manchester, UK; 3 Statistics Unit, QIMR Berghofer Medical Research Institute, Brisbane, Australia; 4 The Netherlands Cancer Institute, Amsterdam, The Netherlands; 5 Institute of Cardiovascular and Cell Sciences (Dermatology Unit), St George’s University of London, London, UK; 6 Faculty of Medicine, The University of Queensland, Brisbane, QLD Australia; 7 Department of Nephrology, University of Queensland at Princess Alexandra Hospital, Brisbane, QLD Australia; 8 Queensland Lung Transplant Service, The Prince Charles Hospital, Brisbane, QLD Australia; 9 School of Clinical Medicine, The University of Queensland, Brisbane, QLD Australia; 10 Dermatology Research Centre, The University of Queensland Diamantina Institute, The University of Queensland, Brisbane, QLD Australia; 11 Department of Dermatology, Princess Alexandra Hospital, Brisbane, QLD Australia

**Keywords:** Cryotherapy, Diathermy, Topical treatment, Skin cancer, Organ transplant recipients

## Abstract

Various treatments of keratotic skin lesions and early skin cancers are performed in organ transplant recipients (OTRs) at high risk of skin malignancies but the frequency of their use is unknown. We prospectively assessed the frequency of use of cryotherapy, diathermy, and topical therapies and also investigated their associations with background incidence of histologically-confirmed squamous-cell carcinoma (SCC) and basal cell carcinoma (BCC) in a cohort of OTRs in Queensland, Australia. Median follow-up ranged from 1.7 to 3.2 years across organ transplant groups. Among 285 kidney, 125 lung and 203 liver transplant recipients [382 (62%) male, 380 (62%) immunosuppressed > 5 years, 394 (64%) previously diagnosed with skin cancer], 306 (50%) reported treatment of skin lesions with major types of non-excision therapies during follow-up: 278 (45%) cryotherapy or diathermy; 121 (20%) topical treatments. Of these 306, 150 (49%) developed SCC at double the incidence of those who did not receive these treatments, as assessed by incidence rate ratio (IRR) adjusted for age, sex, type of organ transplant, skin color and history of skin cancer at baseline, calculated by multivariable Poisson regression (IRR_adj_ = 2.1, 95% confidence interval (CI) 1.4–3.1). BCC incidence was not associated with these therapies. Skin lesions in OTRs that are treated with cryotherapy, diathermy, or topical treatment warrant judicious selection and careful follow-up.

## Introduction

Organ transplantation prolongs life but subsequent immunosuppressant therapy increases development of keratotic skin lesions including skin cancer [3]. Incidence rates of histologically-confirmed cutaneous squamous-cell carcinoma (SCC) are known to be up to 77 times higher in organ transplant recipients (OTRs) than the general population, and rates of basal cell carcinoma (BCC) are 18 times higher [7]. Such rates do not reflect the overall burden of skin tumors in OTRs however as they do not account for the many clinically benign keratotic skin lesions [1, 2] treated destructively or topically.

Various destructive and topical therapies are used in OTRs because, for both the patient and the doctor, they are simpler, less expensive, and more easily carried out than excisions, and expedient when numerous lesions are present and for very large lesions where excision may be impractical [2]. The most common destructive modality is cryotherapy, followed by curettage and electrodessication. Topical treatments like imiquimod cream, diclofenac gel and 5-fluorouracil 5% cream are also popular treatments of malignant and pre-malignant tumors in OTRs [5]. Neither the frequency of use of destructive and topical therapies in OTRs is known, nor is the relationship of these treatments with the background incidence of histologically confirmed SCCs and BCCs. We investigated the scale of use of these therapies in OTRs as an indicator of their burden of pre-malignant and early malignant skin tumor burden and estimated the association with background incidence of SCC and BCC in OTRs so treated.

## Materials and methods

Participants enrolled in the skin tumours in allograft recipients (STAR) study comprised kidney or liver transplant recipients at high-risk of skin cancer (aged ≥ 18 years with past history of skin cancer, or aged ≥ 40 years, or immunosuppressed for ≥ 10 years) and lung transplant recipients, recruited from transplant centers in Brisbane 2012–2015. Institutional ethics committees approved the study and all participants provided written informed consent. At recruitment, OTRs completed self-administered questionnaires about standard skin cancer risk factors including skin color, and past history of skin cancers excised, frozen, or burnt off (hereafter referred to as ‘diathermy’ since performance of curettage was not ascertained). They underwent initial whole-body skin examinations by dermatology-trained physicians who documented the location of clinically-diagnosed skin cancers. Final diagnosis of malignancy was based on histopathological confirmation.

During follow-up for up to 3 years, OTRs underwent annual dermatological examinations for skin cancer and received quarterly phone calls using standard questions to ascertain skin tumor treatments, including cryotherapy, diathermy or topical treatment. Treating physicians also confirmed histologically diagnosed incident cancers they excised, supplemented by regular reviews of pathology laboratories’ databases for newly diagnosed skin cancers in study OTRs.

### Statistical analyses

Quarterly responses regarding destructive/topical treatments were aggregated and summarized as ‘yes/no’ for each modality and overall for the study period. Baseline characteristics associated with these therapies, and with histologically-confirmed SCC or BCC occurring in parallel in the study period were identified in univariate analyses. Associations between destructive or topical treatments and first study SCC/BCC adjusted for significantly associated baseline characteristics, were estimated by incidence rate ratios (IRRs_adj_) and 95% confidence intervals (CIs) calculated by multivariable Poisson regression with offset (person-years at risk) to account for differences of person-time. Person-years at risk was defined as time from baseline skin examination to first SCC/BCC or if no SCC/BCC, to June 30, 2016, date of withdrawal or of death, whichever came first.

## Results

There were 285 kidney, 125 lung and 203 liver transplant recipients (Table 1) with respective median follow-up periods of 3.2, 1.7 and 2.9 years, who participated in quarterly follow-up interviews (382 (62%) male, 380(62%) immunosuppressed > 5 years, 394 (64%) previously diagnosed with skin cancer). During follow-up, 306 (50%) reported treatment of lesions with non-excision therapies of interest: 278 (45%) cryotherapy or diathermy, and 121 (20%) topical treatments. The factors associated with receiving destructive or topical therapies were age > 60 years and fair skin. The proportions receiving cryotherapy or diathermy vs topical treatments did not vary substantially with patients’ characteristics apart from patients treated with cryotherapy or diathermy having more frequent skin checks, and overall, females were proportionately more likely to receive either (Table 1).

**Table 1:** Association of baseline patient characteristics with any destructive/topical, cryotherapy/diathermy and topical treatment during follow-up

Characteristics	Total	Any destructive/topical therapy	Cryotherapy/diathermy therapy	Topical therapy
	*N* = 613	*n* = 306	*n* = 278	*n* = 121
	*N* (%)	*n* (%)	*n* (%)	*n* (%)
Age (years)				
< 50	206 (34)	54 (26)	49 (24)	15 (7)
50–59	178 (29)	96 (54)	86 (48)	37 (21)
60+	229 (37)	156 (68)	143 (63)	69 (30)
Sex				
Female	231 (38)	95 (41)	82 (36)	42 (18)
Male	382 (62)	211 (55)	196 (51)	79 (21)
Type of transplant				
Kidney	285 (47)	150 (53)	133 (47)	73 (26)
Lung	125 (20)	56 (45)	53 (42)	20 (16)
Liver	203 (33)	100 (49)	92 (45)	28 (14)
Years of immunosuppression				
1–5	233 (38)	122 (52)	111 (48)	41 (18)
> 5	380 (62)	184 (48)	167 (47)	80 (21)
Completed education, *n* = 569				
Grade 12 or less	305 (54)	165 (54)	151 (50)	76 (25)
Trade/diploma	154 (27)	72 (47)	63 (41)	23 (15)
University/college	110 (19)	51 (46)	48 (44)	20 (18)
Skin color				
Olive/medium	240 (39)	98 (41)	85 (35)	33 (14)
Fair	373 (61)	208 (56)	193 (52)	88 (24)
Previous skin cancer, *n* = 571				
No	177 (31)	34 (19)	30 (17)	8 (5)
Yes	394 (69)	254 (65)	232 (59)	111 (28)
Frequency of skin checks in last 5 years, *n* = 571				
Less than once/once a year	367 (64)	153 (42)	139 (38)	55 (15)
More than once a year	204 (36)	135 (66)	123 (60)	64 (31)

During follow-up, 189 (31%) developed at least one SCC and 169 (28%) at least one BCC. Of those receiving destructive or topical treatment, 150 (49%) developed SCC; a significant doubling of incidence of SCC compared with those not receiving such treatments (IRR_adj_ = 2.1, 95% CI 1.4–3.1) after adjusting for age, sex, type of organ transplant, skin color and history of skin cancer at baseline (Table 2). The incidence of SCC was also significantly elevated in those who received individual modalities, namely cryotherapy/diathermy (IRR_adj_ = 1.9) and topical treatment (IRR_adj_ = 1.7). However, the incidence of BCC was not elevated among those receiving destructive or topical treatment after adjusting for potential confounding factors (IRR_adj_ = 1.1), whether cryotherapy/diathermy (IRR_adj_ = 1.2) or topical treatment (IRR_adj_ = 1.2) (Table 2).

**Table 2:** Prevalence of receiving destructive/topical therapies and risk of developing SCC and BCC in parallel to destructive/ topical therapy during follow-up

	Total	Developed SCC (*n* = 189)		Developed BCC (*n* = 169)	
	(*n* = 613)	*N* (%)	IRR (95% CI)^a^	*N* (%)	IRR (95% CI)^b^
Destructive treatment					
No	307	39 (13)		46 (15)	
Yes	306	150 (49)	2.1 [1.4–3.1]	123 (40)	1.1 [0.8–1.7]
Cryotherapy/diathermy					
No	335	50 (15)		58 (17)	
Yes	278	139 (50)	1.9 [1.3–2.7]	111 (40)	1.2 [0.9–1.7]
Topical treatment					
No	492	116 (24)		109 (22)	
Yes	121	73 (60)	1.7 [1.2–2.3]	60 (50)	1.2 [0.9–1.7]

*SCC* squamous cell carcinoma, *BCC* basal cell carcinoma, *IRR* incidence rate ratio, *CI* confidence interval

^a^Adjusted for age, sex, type of transplant, skin color and history of skin cancers excised/treated (Nil, ≤ 5, 6–10 and > 10)

^b^Adjusted for age, sex, type of transplant, education and history of skin cancers excised/treated (Nil, ≤ 5, 6–10 and > 10)

## Discussion

We found that 50% OTRs reported having destructive or topical therapies during follow-up of at least 1 year. This high proportion varied little by organ transplanted but varied directly with skin cancer risk factors. Cryotherapy/diathermy was used twice as frequently as topical treatment, and both groups of treatment were used proportionately more in female than male OTRs. There are no known comparable studies in OTRs: the few data available regarding prevalence of these non-excisional treatments have been based on registers of medical insurance claims in the general population [6]. The major limitation of our study in a specific population of high-risk OTRs was that it was based on prospective self-reports of treatment by the patients every 3 months, but this was the only feasible method to obtain such data from the sizable study cohort. The results are consistent with extreme morbidity due to skin tumors upstream from the frank skin malignancies known to affect these patients. The very high frequency of non-excisional therapies also signifies an undocumented heavy use of dermatologic services beyond skin cancer treatments, with one in two high-risk OTRs receiving cryotherapy/diathermy or topical treatments during relatively short-term follow-up. Many of these OTRs have severe and widespread actinic damage [4] with numerous keratoses and skin cancers [7], and thus minimally invasive approaches are often preferable to treatment by excision.

As anticipated, the incidence of SCC in the study period was significantly elevated in those who were receiving non-surgical treatments of skin lesions, but surprisingly the incidence of BCC was not, and the reasons for this discrepancy are unclear since destructive and topical therapies were viewed a priori as likely markers of high risk of both SCC and BCC [1]. In addition both non-aggressive BCCs and early SCCs may have been treated without surgery since many of these patients had extensive actinic field change [5] and differentiating skin cancers from benign keratotic tumors in OTRs is often difficult [1]. The lack of association with BCC may reflect a much lower use of cryotherapy/diathermy and topical treatments on sites like the trunk and lower limbs that are more commonly affected by BCC than SCC [4], but we did not collect information about actual sites of cryotherapy or topical treatment.

In conclusion, these results show extensive use of cryotherapy/diathermy and topical treatments in OTRs at high risk of skin cancer. They also suggest that judicious use of these treatments in OTRs is called for, since they are strongly related to risk of SCC. They also suggest that ongoing intensive surveillance for invasive SCC is warranted in this patient group.
